# Supplementary material for: Getting More Out of Biomedical Documents with GATE's Full Lifecycle Open Source Text Analytics
Source: PLoS Comput Biol. 2013 Feb 7;9(2):e1002854. doi: 10.1371/journal.pcbi.1002854 (PMC3567135; doi:10.1371/journal.pcbi.1002854)
Supplement: Dataset S1 — GATE software. Dataset S1 bundled with this paper contains a distribution of GATE (or see http://gate.ac.uk/download/). (TGZ) [file pcbi.1002854.s001.tgz › plos-gate/plugins/Lang_Chinese/resources/segmenter/segmenter.html]

Chinese Segmenter and Annotation Tool

You can download the zip file segment.zip which
contains four files. First is the perl script segment.pl which takes
one argument, the name of the source file to segment. It expects the
file name to end with ".txt". It needs the library file segmenter.pl
which has all the actual segmenation code. The program also expects
to find the lexicon file wordlist.txt in the same directory it's
running in (though this is easily modified). It outputs a new
segmented file with ".txt" replaced with ".seg". Right now it only
works on GB encoded files, but a Big5 version (converting to GB,
segmenting, and using the segmented file to segment the original Big5
version file) would not be hard. Also included is a convenience file,
segment.bat, for people working in Windows. It runs perl on
segment.pl and expects a file name as an argument.

The segmenter requires Perl to run.
It is a free and easily downloaded program.

I have also made available a Java version
of the segmenter that works with Big5, GB, and UTF-8 encoded
text files.
> Usage: java -jar segmenter.jar [-b|-g|-8] inputfile.txt  
> -b Big5, -g GB2312, -8 UTF-8  
> Segmented text will be saved to inputfile.txt.seg

Words can be added or deleted directly from the lexicon file. The
segmenter has algorithms for grouping together the characters in a name,
especially for Chinese and Western names, but Japanese and South-east Asian
names may not work well yet.

The segmentation process is also a perfect time to identify interesting
"entities" in the text. These could include dates, times, person names,
locations, money amounts, organization names, and percentages. This
collection of interesting nouns is often refered to as "named entities"
and the process of identifying them as "named entity extraction". There
is already code to identify person names and number amounts in the segmenter,
and I will adding more code to find the rest in the future.

The segmenter works with a version of the maximal matching algorithm.
When looking for words, it attempts to match the longest word possible.
This simple algorithm is suprisingly effective, given a large and diverse
lexicon, but there also need to be ways of dealing with ambiguous word
divisions, unkown proper names, and other words not in the lexicon. I
currently have algorithms for finding names, and am researching ways to
better handle ambiguous word boundaries and unknown words. Additional
knowledge that would be useful would be a list of characters and whether
they are bound or unbound. A segmentation that would leave a bound character
by itself would not be allowed. A statistical way of choosing amongst
ambiguous segmentations would also be useful.

More information on segmenting Chinese text can be found at
ChineseComputing.com.

Contact Erik Peterson at erik@mandarintools.com
with questions or comments. Please visit 
Online Chinese Tools for many more useful Chinese-related software tools.
